# Atrial Natriuretic Peptide Improves Neurite Outgrowth from Spiral Ganglion Neurons *In Vitro* through a cGMP-Dependent Manner

**DOI:** 10.1155/2020/8831735

**Published:** 2020-08-28

**Authors:** Fei Sun, Ke Zhou, Ke-yong Tian, Jie Wang, Jian-hua Qiu, Ding-jun Zha

**Affiliations:** ^1^Department of Otolaryngology-Head and Neck Surgery, Xijing Hospital, Fourth Military Medical University, Xi'an, Shaanxi 710032, China; ^2^Center of Clinical Laboratory Medicine of PLA, Department of Laboratory Medicine, Xijing Hospital, Fourth Military Medical University, Xi'an, Shaanxi 710032, China; ^3^Department of Otolaryngology-Head and Neck Surgery, The Affiliated Children Hospital of Xi'an Jiaotong University, Xi'an, Shaanxi 710003, China

## Abstract

The spiral ganglion neurons (SGNs) are the primary afferent neurons in the spiral ganglion (SG), while their degeneration or loss would cause sensorineural hearing loss. As a cardiac-derived hormone, atrial natriuretic peptide (ANP) plays a critical role in cardiovascular homeostasis through binding to its functional receptors (NPR-A and NPR-C). ANP and its receptors are widely expressed in the mammalian nervous system where they could be implicated in the regulation of multiple neural functions. Although previous studies have provided direct evidence for the presence of ANP and its functional receptors in the inner ear, their presence within the cochlear SG and their regulatory roles during auditory neurotransmission and development remain largely unknown. Based on our previous findings, we investigated the expression patterns of ANP and its receptors in the cochlear SG and dissociated SGNs and determined the influence of ANP on neurite outgrowth *in vitro* by using organotypic SG explants and dissociated SGN cultures from postnatal rats. We have demonstrated that ANP and its receptors are expressed in neurons within the cochlear SG of postnatal rat, while ANP may promote neurite outgrowth of SGNs via the NPR-A/cGMP/PKG pathway in a dose-dependent manner. These results indicate that ANP would play a role in normal neuritogenesis of SGN during cochlear development and represents a potential therapeutic candidate to enhance regeneration and regrowth of SGN neurites.

## 1. Introduction

Sensorineural hearing loss (SNHL) is a major health problem which affects millions of individuals worldwide. SNHL is associated with irreversible degeneration of the cochlear sensory cells within the auditory portion of the inner ear, including hair cells (HCs) and spiral ganglion neurons (SGNs). In the mammalian inner ear, the HCs in the organ of Corti function in transducing the sound mechanical stimulation into the primary acoustic signals [1–3], while the SGNs are the primary afferent neurons in the spiral ganglion (SG) and play a critical role in hearing, transmitting primary acoustic information from HCs to the higher auditory centers of the central nervous system (CNS) [4–6]. Loss of HCs, primarily resulting from noise trauma, ototoxic drugs, infections, aging, and genetic mutations, with sequential degeneration of SGNs, ultimately leads to permanent SNHL [7–15]. The current preferred treatment of SNHL for patients with profound HC loss and mostly intact SGNs includes cochlear implants, which uses electrode arrays to substitute for mechanosensory HCs in generating electrical impulses to the auditory nerve [16]. In order to promote regeneration and guiding of neurites from residual auditory neurons, many potential guidance cues are under research, due to their influence on neurite outgrowth behavior and subsequent performance of cochlear implants [6, 17–23].

SG is a peripheral cluster of both neurons and glial cells located in Rosenthal's canal, which coils around the cochlear modiolus and forms the auditory nerve. SGNs are divided into two subpopulations, type I and type II, according to their different morphologies, synaptic connections, and functions. Approximately 95% of SGNs are larger, bipolar, and myelinated type I neurons, which can be further subdivided into three subtypes (Type IA, Type IB, and Type IC), innervating the inner HCs with their peripheral dendrites to principally encode the auditory signals [24–26]. The remaining 5% of SGNs are smaller, pseudomonopolar, and nonmyelinated type II neurons, which innervate the outer HCs and some of the supporting cells to provide sensory feedback, controlling the sensitivity of the auditory epithelium to specific sound stimuli. Additionally, the perikaryons of all types of SGNs are enveloped by satellite glial cells, forming loose myelin around the type I neuronal somata. Both peripheral dendrites and intracochlear axons of type I SGNs are myelinated by Schwann cells, whereas their central axons from the peripheral-central glial transition zone (glia limitans) to the terminal synapses in the cochlear nucleus are myelinated by oligodendrocytes and astrocytes [27, 28]. It is necessary to understand the expression, function, and signaling interactions of the regulatory substances which affect axonal development and neuronal plasticity of primary auditory neurons, to offer optimal strategies of manipulating connections between sensory epithelium or implanted electrodes and neurites of SGNs, and eventually provide promising pharmacological targets that facilitate new and effective therapies for hearing impairment.

Atrial natriuretic peptide (ANP) is a 28 amino acid peptide predominantly synthesized and secreted by the cardiac atria and is the first member of the natriuretic peptide family [29], which also includes brain natriuretic peptide (BNP) and C-type natriuretic peptide (CNP). ANP interacts with two specific, high affinity natriuretic peptide receptors, NPR-A and NPR-C, on the plasma membrane of target cells to mediate its physiological effects [30–32]. The natriuretic peptide receptor-A (NPR-A, also known as NPR1 or GC-A) is a transmembrane receptor coupled to the particulate guanylyl cyclase (GC) which catalyzes the synthesis of the second messenger cyclic guanosine-3′,5′-monophosphate (cGMP). cGMP modulates the activity of specific effector molecules including cGMP-regulated isoforms of phosphodiesterases, cyclic-nucleotide-gated ion channels, and cGMP-dependent protein kinases G (PKG), which in turn regulate diverse biological responses associated with blood vessel tone, transepithelial ion transportation, neuronal excitability, neuronal development, and neurite pathfinding, and the sensory transduction pathways underlying olfaction and vision [31, 32]. The natriuretic peptide receptor-C (NPR-C), which lacks the GC domain, contributes to the clearance of ANP and other natriuretic peptides from the circulation through receptor-mediated internalization and degradation. In addition, evidence has shown that NPR-C can also affect other second messenger signaling by activating phospholipase C and inhibiting adenylyl cyclase [33].

In addition to the cardiovascular system, the tissue-specific distribution and function of ANP, NPR-A, and NPR-C have been established in several tissues including the kidney, adrenal, lung, adipose tissue, and retina. Furthermore, ANP and its receptors have been found in the CNS, leading us to speculate that ANP may function as a neuromodulator or neuropeptide involved in neuronal and glial functions [34–36]. Importantly, their presence in the secretory and sensory compartments of the rodent inner ear is well documented, suggesting ANP may act as a local hormone regulating the fluid and electrolyte balance in the inner ear [37–51]. Previous reports revealed that ANP receptors have been localized to the cochlear modiolus of the guinea pig [42] and rat SG [51]. However, little is known regarding the localization and functional roles of ANP and its receptors in the inner ear, and here, we have focused our attention on them.

In our previous study, we have already investigated the expression patterns of ANP and its receptors, which provided direct evidence for the presence and synthesis of ANP as well as its receptors in the cochlear SG [52, 53]. In our current study, we reassessed the distribution of ANP and its receptors in the cochlear SG as well as in dissociated SGNs, and determined the influence of ANP on neurite outgrowth *in vitro* by using organotypic SG explants and dissociated SGN cultures from postnatal rats. We have demonstrated that ANP and its receptors are expressed in neurons within the cochlear SG of postnatal rat, while ANP may promote neurite outgrowth of SGNs via the NPR-A/cGMP/PKG pathway in a dose-dependent manner.

## 2. Materials and Methods

### 2.1. Animals and Tissue Preparation

All experiments were approved by the Animal Care Committee of Fourth Military Medical University, China, on the care and use of Laboratory Animal for Research Purposes. All cochleae used in this investigation were obtained from postnatal day 3 (P3) or day 14 (P14) Sprague-Dawley rats provided by the Laboratory Animal Center of the Fourth Military Medical University. All rat pups were sacrificed by decapitation, and the skulls were opened midsagitally. With the aid of a dissecting microscope (SZX16; Olympus, Japan), the rat cochleae were removed from the temporal bone, washed in ice-cold Hank's Balanced Salt Solution (HBSS; Thermo Fisher Scientific, USA), and collected for further use.

### 2.2. Preparation of Cochlear Sections and Spiral Ganglion Neurons Culture

For cochlear cryosections, the cochleae from P14 rats were fixed with 4% paraformaldehyde (PFA) in phosphate buffer (PB; 0.1 M, pH 7.2) by perfusion via the round and oval windows and then incubated with the same fixative overnight at 4°C. The cochleae were decalcified in a 5% EDTA solution for 2 days, followed by cryoprotection in 30% sucrose solution overnight at 4°C. The samples were then embedded in Tissue-Tek OCT compound (Sakura Finetek, USA) at -20°C, sectioned into 12 *μ*m thick midmodiolar cross-sections using a cryostat microtome (CM1850; Leica, Germany) and mounted on poly-L-lysine-coated slides.

Dissociated cultures of SGNs were prepared from P3 rat pups and maintained as described previously [54–56]. Briefly, each SG was isolated from the cochlea in ice-cold HBSS by sequential removal of the bony cochlear capsule, the spiral ligament, and the organ of the Corti, leaving the SGNs within the modiolus. The modiolus tissues were transferred into Ca^2+^/Mg^2+^ free HBSS with 0.25% trypsin and 0.1% collagenase type IV (all Thermo Fisher Scientific) at 37°C for 20 min to enzymatically dissociate the cells. The enzymatic reaction was quenched by the addition of 10% fetal bovine serum (FBS; Thermo Fisher Scientific). After three washes with culture medium, the tissues were mechanically dissociated by trituration with a fame-polished Pasteur pipette. The dissociated cells were resuspended in a neural maintenance medium consisting of Dulbecco's modified Eagle medium/Ham's F12 medium (DMEM/F12) supplemented with 1x B27, 1x N2, and 1% penicillin-streptomycin (all Thermo Fisher Scientific) and plated at a density of 1.0 × 10^6^ cells/glass bottom dish previously coated with poly-L-lysine (0.1 mg/mL in 10 mM borate buffer, pH 8.4; Thermo Fisher Scientific) to adhere for 4 h at 37°C, 5% CO_2_, and 95% humidity. After attachment was confirmed under an inverted microscope (Eclipse TE2000-U; Nikon, Japan), 1 mL of neural maintenance medium was added in each neuronal cell culture and incubated for 48 h prior to fixation with 4% PFA for 20 min at room temperature (RT).

### 2.3. Expression Pattern Analysis of ANP and Its Receptors by Immunofluorescence

For immunohistochemistry, the cochlear sections and fixed SGNs cultures were washed with phosphate-buffered saline (PBS; 0.01 M, pH 7.4), blocked with 5% bovine serum albumin (BSA; Sigma-Aldrich, USA) and 0.1% Triton X-100 in PBS for 40 min at 37°C, and incubated overnight at 4°C with the following primary antibodies diluted in antibody solution (1% BSA and 0.1% Triton X-100 in PBS): polyclonal rabbit anti-ANP antibody (1 : 500; Cat# PA5-29559, Thermo Fisher Scientific), polyclonal rabbit anti-NPR-A antibody (1 : 500; Cat# PA5-29049, Thermo Fisher Scientific), polyclonal rabbit anti-NPR-C antibody (1 : 500; Cat# PA5-96947, Thermo Fisher Scientific), and monoclonal mouse anti-Tubulin *β*-III (TUJ1) antibody (1 : 500; Cat# ab78078, Abcam, UK). After washing, samples were treated with the appropriate secondary antibodies diluted in antibody solution for 2 h at RT: Alexa Fluor 488-conjugated donkey anti-mouse IgG (1 : 500; Cat# A-21202, Thermo Fisher Scientific) and Alexa Fluor 594-conjugated donkey anti-rabbit IgG (1 : 500; Cat# A-21207, Thermo Fisher Scientific). Each experiment also included a negative control where the primary antibody was omitted. After rinsing, specimens were treated with the nuclear stain, 4′,6-diamidino-2-phenylindole (DAPI; diluted 1 : 1000; Thermo Fisher Scientific) for 15 min at RT, mounted with Prolong Gold anti-fading mounting medium (Thermo Fisher Scientific), and subsequently examined under a spectral scanning laser confocal microscope (FV1000; Olympus, Japan). All images were saved as TIFF files using Olympus confocal software (FV10-ASW 4.2; Olympus) and processed with Adobe Photoshop CS6 (Adobe Systems, USA) for adjustments of brightness and/or contrast.

### 2.4. Spiral Ganglion Explants and Spiral Ganglion Neuron Cultures

The cochlea dissection procedures were performed as described in previous studies with slight modifications [56–59]. The cochleae from P3 rats were immersed in ice-cold HBSS; then, the cochlear capsule was opened by fine forceps and the membranous labyrinth was removed from the modiolus under a dissecting microscope. The spiral lamina containing the SG was carefully separated from the modiolus and cut into equal portions of 300~500 *μ*m before being transferred to the culture dish. The Cell-Tak Cell and Tissue Adhesive (Corning, USA) precoated 15 mm glass bottom culture dishes (Advance BioScience, USA) were loaded with 100 *μ*L primary attachment medium consisting of DMEM, 10% FBS, 25 mM HEPES buffer, and 1% penicillin-streptomycin (all Thermo Fisher Scientific). Then, each dissected explant was plated onto single-glass bottom dish, and the culture medium was carefully aspirated from dishes containing SG explants, leaving only 10 *μ*L to allow the tissue to settle for 3 ~ 5 min. For three-dimensional culture, 100 *μ*L of a 20% Matrigel (Corning) mixture diluted in the primary attachment medium was dropped on to the tissue explants directly and left the tissues adhering overnight at 37°C, 5% CO_2_, and 95% humidity. After attachment was confirmed, SG explants incubated in neural maintenance medium with or without 20 ng/mL recombinant brain-derived neurotrophic factor (BDNF; PeproTech, USA) were served as control cultures. Experimental cultures were incubated in neural maintenance medium supplemented with 100 nM or 1 *μ*M of ANP (Caymanchem, USA), 1 *μ*M of the membrane-permeable cGMP analogue 8-(4-chlorophenylthio) guanosine-3′,5′-cyclic monophosphate (8-pCPT-cGMP; Sigma-Aldrich), or 1 *μ*M ANP plus 1 *μ*M of the PKG inhibitor KT5823 (Sigma-Aldrich), respectively. For each condition, three cochlear neural explants were cultured for 7 days in a 37°C humidified incubator containing 5% CO_2_ prior to fixation for neurite outgrowth study, and culture medium was changed every other day.

The dissociated SGNs prepared as described above, were resuspended in neural maintenance medium and plated at a density of 2.0 × 10^5^ cells/coated glass bottom dish. After attachment, cells from different experimental cultures were incubated in neural maintenance medium in the absence or in the presence of pharmacological reagents identical to those in SG explant cultures: 20 ng/mL BDNF, 100 nM ANP, 1 *μ*M ANP, 1 *μ*M 8-pCPT-cGMP, or 1 *μ*M ANP plus 1 *μ*M KT5823. For each condition, three culture dishes seeded with dissociated SGNs were fed with fresh medium every other day and cultured for 5 days prior to fixation.

### 2.5. Immunofluorescent Analysis of Neurite Outgrowth Spiral Ganglion Explants and Neurons

After the culture period, SG explants or SGN cells were fixed in 4% PFA for 20 min at RT. Both explants and cells were blocked with 5% BSA and 0.1% Triton X-100 in PBS, followed by incubation with anti-Tubulin *β*-III primary antibody (diluted 1 : 500) and Alexa Fluor 488-conjugated donkey anti-mouse IgG (diluted 1 : 500) to selectively stain the neural components of the explants or cells, then counterstained with DAPI (diluted 1 : 1000) to visualize the nuclei. All specimens in the glass bottom dish were mounted with Prolong Gold medium and examined under the confocal microscope.

*In vitro* Images of the immunostained cultures were analyzed by using ImageJ software (version 1.46r; NIH, USA) according to a previous study [60]. Neurite tracing was performed by using the “Analyze-Set Scale” function, the pixel unit of neurite length measurement was set in micrometers. Images were rendered with segmentation function, and neurite tracer function was applied choosing the starting point at the SGN cell bodies, resulting in a compiled skeleton render of all measured neurites. Only those neurites contained entirely within the image were analyzed. Neurite outgrowth from the SG explants was evaluated by measuring the number and lengths of the processes. Total number and the neurite length of the dissociated SGNs were also analyzed. Statistical analysis was performed using a one-way analysis of variance (ANOVA) followed by Bonferroni's post hoc test. Data presented in the text and figures are means and standard error of the mean (means ± SEM). Analysis was performed using the Statistical Program for Social Science software (SPSS, version 22.0; IBM Inc., USA). *P* values less than 0.05 (*P* < 0.05) were considered statistically significant.

## 3. Results

### 3.1. Distribution of ANP and Its Receptors in Spiral Ganglion and Spiral Ganglion Neurons of Postnatal Rats

**Figure 1:**
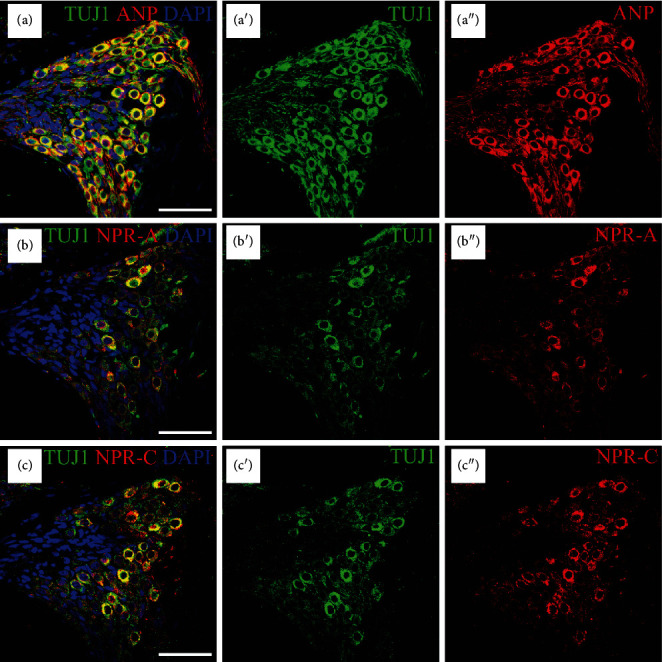
Immunolocalization of ANP, NPR-A, and NPR-C in SGNs within the SG of P14 rats. Merge and single-channel images of cochlear sections triple labeled with antibodies against neural marker TUJ1 (green), ANP/NPR-A/NPR-C (red), and DAPI (blue). (a) ANP was predominantly immunoreactive in the perikarya of SGNs. NPR-A (b) and NPR-C (c) were predominantly immunoreactive in the plasma membrane and cytoplasm of SGNs and appeared more pronounced in the cellular membrane. Scale bars = 50 *μ*m.

To investigate the localizations of ANP and its receptors within the SG, we coimmunostained cochlear sections from P14 rats with antibodies against a neuronal marker, class III *β*-tubulin. No noticeable apical-to-basal gradients of ANP and its receptors immunoreactivities were observed in the SG regions along the length of the cochlear tonotopic axis. Hence, the midcochlear turn was taken as a representative of the entire length of the cochlea for analysis of the cellular localizations of ANP and its receptors. As shown in Figure 1, the immunoreactivity of ANP, NPR-A, or NPR-C was colocalized with *β*-III tubulin-positive somata of SGNs, respectively. ANP was predominantly immunoreactive in the neuronal perikarya, including the plasma membrane and cytoplasm of SGNs (Figure 1(a)). The distribution of NPR-A (Figure 1(b)) and NPR-C (Figure 1(c)) in SGNs was rather similar, and they were predominantly immunoreactive in the plasma membrane and cytoplasm of SGNs and appeared more pronounced in the cellular membrane. Some heterogeneity in the levels of immunoreactivities of ANP, NPR-A, and NPR-C were also observed, with less colocalization in a subpopulation of SGNs. No immunoreactivity was observed in the negative controls where the primary antibody was omitted (data not shown).

**Figure 2:**
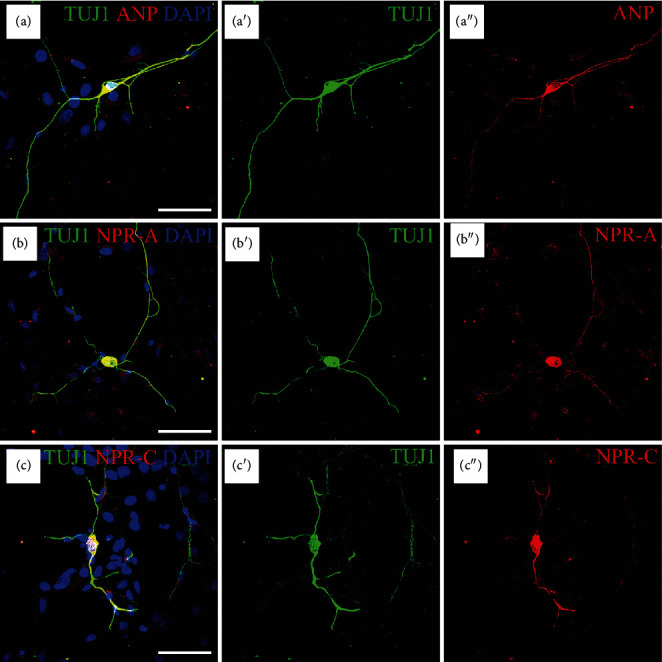
Immunolocalization of ANP, NPR-A, and NPR-C in dissociated SGNs from P3 rats. Merge and single-channel images of SG cells triple labeled with antibodies against TUJ1 (green), ANP/NPR-A/NPR-C (red), and DAPI (blue). The immunoreactivity of ANP (a), NPR-A (b), or NPR-C (c) was colocalized with *β*-III tubulin-positive SGNs, respectively, distributed in the neuronal soma and neurites. Scale bars = 50 *μ*m.

To confirm the distribution pattern of ANP and its receptors in SGNs, we also performed immunohistochemistry on SG cell cultures using the same neuronal marker. As shown in Figure 2, the immunoreactivity of ANP, NPR-A, or NPR-C was colocalized with *β*-III tubulin-positive SGNs, respectively. In detail, the distribution of ANP and its receptors were immunoreactive all over each neuron including soma and neurites. No immunoreactivity was also observed in the negative controls where the primary antibody was omitted (data not shown).

### 3.2. Influence of ANP on Neurite Number and Neurite Length in Spiral Ganglion Explants

**Figure 3:**
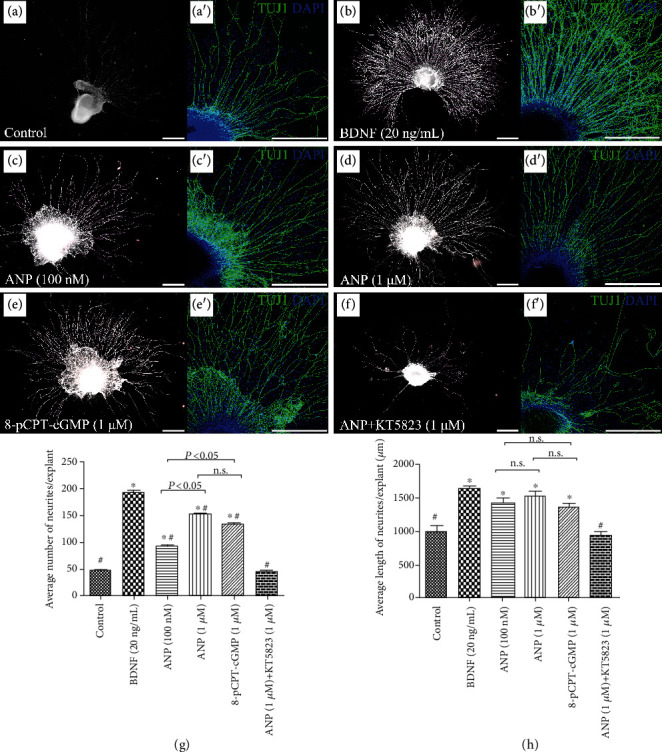
ANP promotes neurite outgrowth of SG explants *in vitro*. Immunohistochemical analysis of cochlear SG explants maintained in culture medium alone as a control (a), or treated with 20 ng/mL BDNF (b), 100 nM ANP (c), 1 *μ*M ANP (d), 1 *μ*M 8-pCPT-cGMP (e), or 1 *μ*M ANP plus 1 *μ*M KT5823 (f) for 7 d. Neurons were labeled with TuJ1 (green), while nuclei were stained with DAPI (blue). Scale bars = 500 *μ*m. In each experimental culture, three cochlear neural explants were studied for neurite outgrowth, and the number of neurite outgrowth (g) and average neurite outgrowth length (h) of explants were calculated. Results are expressed as mean ± SEM (^∗^*P* < 0.05, versus negative control samples; ^#^*P* < 0.05, versus positive control samples/BDNF; n.s., *P* > 0.05).

To determine the possible role of ANP in affecting neurite outgrowth of SGNs, we firstly quantified the number and length of neurites from SG explants from P3 rats maintained in culture medium supplemented with different reagents *in vitro* for 7 d. Representative images from different experimental cultures were shown in Figure 3. The SG explants incubated in culture medium without any supplement were used as a negative/baseline control, while explants incubated in culture medium supplemented with BDNF for trophic support of neurite outgrowth of SGNs were served as a positive control. Immunofluorescence and quantitative analysis of neuron-specific *β*-III tubulin-positive neurites revealed the number of neurites per explant was 46.0 ± 3.8, and the average neurite length was 1002.8 ± 155.5 *μ*m for negative control samples (Figure 3(a)). As expected, abundant neurite sprouting and elongating were seen in explants treated with 20 ng/mL BDNF, and the average number and length of neurites from positive control samples were 191.7 ± 5.8 and 1645.8 ± 58.1 *μ*m, respectively (Figure 3(b)). Strikingly, robust neurite extension from SG explants was seen after treatment with different dosage of ANP. The number and length of neurites were 92.0 ± 1.7 and 1438.3 ± 141.0 *μ*m for 100 nM ANP-treated samples (Figure 3(c)) and were 151.0 ± 2.3 and 1551.2 ± 109.1 *μ*m for 1 *μ*M ANP-treated samples (Figure 3(d)), respectively. At any dosage of ANP (100 nM and 1 *μ*M), a statistically significant increase in neurite outgrowth compared to the negative control was observed, while a significant difference was also found when compared to the positive control. To gain insight into the mechanism of ANP in promoting neurite outgrowth, we investigated whether this peptide acts through the GC-coupled receptors, NPR-A, which could elicit cGMP production. Results indicated that number (133.0 ± 3.79) and length (1374.8 ± 102.5 *μ*m) of elongating neurites from SG explants treated with 1 *μ*M 8-pCPT-cGMP, a cGMP analogue, were also significantly different from the negative and positive controls (Figure 3(e)). Treatment with 1 *μ*M KT5823, a selective inhibitor of PKG, appeared to abrogate SGN neurite sprouting and outgrowth (47.3 ± 5.2 and 955.2 ± 109.2 *μ*m) in the presence of 1 *μ*M ANP (Figure 3(f)), and the resulting neurite outgrowth did not significantly differ from the negative control. Taken together, these results indicated that ANP could promote SG neurite outgrowth via the NPR-A/cGMP/PKG pathway in a dose-dependent manner.

### 3.3. Influence of ANP on Cell Number and Neurite Length of Dissociated Spiral Ganglion Neurons

**Figure 4:**
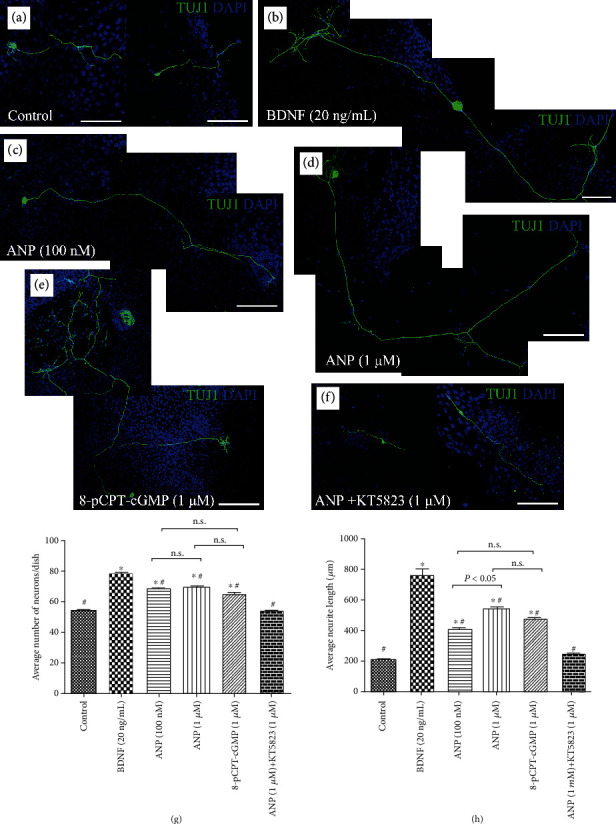
ANP promotes neurite outgrowth of dissociated SGNs *in vitro*. Immunohistochemical analysis of dissociated SGNs maintained in culture medium alone (a), or treated with 20 ng/mL BDNF (b), 100 nM ANP (c), 1 *μ*M ANP (d), 1 *μ*M 8-pCPT-cGMP (e), or 1 *μ*M ANP plus 1 *μ*M KT5823 (f) for 5 d. Neurons were labeled with TuJ1 (green), while nuclei were stained with DAPI (blue). Scale bars = 100 *μ*m. In each experimental culture, three SGNs seeded-culture dishes were studied for neurite outgrowth, and the cell number (g) and average neurite outgrowth length (h) of dissociated SGNs were calculated. Results are expressed as mean ± SEM (^∗^*P* < 0.05, versus negative control samples; ^#^*P* < 0.05, versus positive control samples/BDNF; n.s., *P* > 0.05).

To validate the influence of ANP on the survival and neurite outgrowth of SGNs, we subsequently quantified the cell number and neurite length of dissociated SGNs from P3 rats maintained in culture medium with additives identical to those in explant cultures *in vitro* for 5 d. The dissociated neurons incubated in culture medium supplemented with or without 20 ng/mL BDNF were used as a positive or negative control, respectively. Representative images from different experimental cultures were shown in Figure 4. The average number of neurons per culture dish was 54.0 ± 1.5, and the average neurite length per neuron was 209.5 ± 19.5 *μ*m in negative control samples (Figure 4(a)). Significantly increased number of neurons (78.3 ± 2.0) and elongating neurite outgrowth (763.2 ± 84.4 *μ*m) were seen in cell cultures treated with 20 ng/mL BDNF (Figure 4(b)). The number of neurons and length of neurite were 69.0 ± 1.2 and 410.4 ± 29.5 *μ*m for 100 nM ANP-treated neurons (Figure 4(c)), 70.0 ± 1.3 and 543.5 ± 31.5 *μ*m for 1 *μ*M ANP-treated neurons (Figure 4(d)), and 65.7 ± 2.9 and 475.2 ± 33.4 *μ*m for 1 *μ*M 8-pCPT-cGMP-treated neurons (Figure 4(e)), respectively. These observations were all significantly different from either negative or positive control samples. As expected, 1 *μ*M ANP failed to either increase neuronal number or induce neurite outgrowth of SGNs (53.6 ± 1.2 and 255.5 ± 18.7 *μ*m) in the presence of 1 *μ*M KT5823 (Figure 4(f)). Collectively, these results indicated that the ANP/NPR-A/cGMP/PKG pathway may promote neuronal survival to some extent and would enhance neurite outgrowth of SGNs in a dose-dependent manner.

Interestingly, when treated with ANP, a number of dissociated SGNs made more than one branches from a single neurite. This effect could be apparently seen in BDNF, ANP, and cGMP analogue 8-pCPT-cGMP-treated neurons, indicating that neurotrophins and ANP signaling could exert a trophic support for neurite outgrowth and axon branching (Figures 4(b)–4(e)). Thus, the effect of axonal branching or pathfinding mediated by the ANP/NPR-A/cGMP/PKG pathway still need more comprehensive evaluation during cochlear development for better understanding the exquisite mechanisms underlying the assembly of auditory circuits.

## 4. Discussion

To promote the regeneration of SGN and to guide the neurite outgrowth of SGN is a critical scientific question in the hearing research fields. In recent years, many previous reports used transcription regulation, biomaterials, electrical stimulation, and magnetic regulation to promote the regeneration and maturation of SGNs and other nervous tissues [23, 57, 60–67]. In our previous researches, we have already studied the expression patterns of ANP and its receptors by immunohistochemical and molecular biological analyses, with the aim of identifying their cellular localizations, expression levels, and the potential functions within the SG of postnatal rat. The immunoreactivity of ANP and its receptors distributed in SGNs and perineuronal glial cells, with differential expression levels of both mRNA and protein in the rat SG during postnatal development. All these data provided a general idea about the temporal and spatial expression profiles of ANP and its receptors within the cochlear SG, suggesting possible roles for ANP in modulating neuronal and glial functions within the SG during auditory neurotransmission and development [52, 53].

In our current study, we performed immunofluorescent analysis on cochlear cryosections as well as dissociated SGNs to validate the distribution of ANP and its receptors in SGNs. Additionally, the influence of ANP on neurite outgrowth was also determined by using organotypic SG explants and dissociated SGN cultures from postnatal rats *in vitro*. We have demonstrated that ANP and its receptors localize in SGNs within cochlear sections and dissociated cultures. ANP, NPR-A, and NPR-C were predominantly immunoreactive in the neuronal perikarya of SGNs, which was consistent with our previous results [52, 53].

ANP, as well as the other two major components of the natriuretic peptide family, BNP and CNP, together with their receptors (NPRs), are widely expressed in the neuronal and glial elements within rodent and mammalian CNS. Additionally, circumstantial evidences indicate that natriuretic peptides and receptors are also distributed in the neural region of peripheral sensory organs, particularly in the sensory ganglia including the dorsal root ganglion (DRG), as well as the ganglia in the retina and inner ear [42, 51, 68–73]. All they could potentially be involved in the regulation of several aspects of neuronal functions such as synaptic transmission and information processing, neural development, neuroprotection, neuroinflammation, neurovascular and blood-brain barrier integrity, and brain fluid homeostasis [34–36]. Especially, many recent researches have shown the significance of the cGMP signaling pathway for neuronal development and neurite pathfinding both in the central and peripheral nervous system, thus providing some hints on the possible functions of natriuretic peptides in axonal development. cGMP signaling elicited by activation of the transmembrane GC-coupled natriuretic peptide receptor NPR-B (also known as NPR2 or GC-B) by the ligand CNP control sensory axon bifurcation of DRG neurons entering the spinal cord [74–83]. Interestingly, the same phenomenon was also found in the cranial sensory ganglion neurons entering the hindbrain including cochlear SGNs [84–88]. Likewise, the mouse lacking cGMP-dependent protein kinase 1 (PKG1) or NPR-B has a defect in the central axonal projection of the DRG sensory neurons [74–78, 81–83] or SGNs [84–88], respectively. Consequently, all these results emphasize a strong significance of the CNP/NPR-B/cGMP/PKG1 pathway regulating neurite outgrowth or pathfinding during neuronal development.

Taking together the results of previous studies and our current work demonstrating colocalization of ANP and its receptors in cochlear SGNs, we hypothesize that ANP may be involved in the regulation of axonal development in the auditory circuits, since ANP also activates a cGMP-dependent signaling cascade upon binding to NPR-A. To test this hypothesis, the influence of ANP on neurite outgrowth was determined in organotypic SG explants and dissociated SGN cultures from postnatal rats *in vitro*. Since neurotrophins play a critical role in SGN development and maintenance and have been shown to promote SGN survival and enhance neurite elongation [89, 90], SG explants and dissociated SGN cultures incubated with BDNF additive were used as a positive control. Without any additional trophic support, neurite outgrowth from SGNs from explants or dissociated cultures in negative control samples is very limited. The observed neurite outgrowth induced by ANP is dose dependent and significantly different from that of negative control cultures. Furthermore, additional experiments using cell permeable cGMP analogue or a PKG inhibitor further confirmed the role of the ANP/NPRA/cGMP/PKG pathway in neurite elongation, since this activity can be replicated by using 8-pCPT-cGMP and abolished by KT-5823. Taken together, all our data indicated that ANP could enhance neurite outgrowth of SGNs via the NPR-A/cGMP/PKG pathway in a dose-dependent manner and may promote neuronal survival to some extent.

In conclusion, we have demonstrated that ANP and its receptors are expressed in neurons within the cochlear SG of postnatal rat, while ANP may promote neurite outgrowth of SGNs via the NPR-A/cGMP/PKG pathway in a dose-dependent manner. These results indicate that ANP would play a role in normal neuritogenesis of SGN during cochlear development and represents a potential therapeutic candidate to enhance regeneration and regrowth of SGN neurites. Our data offer a good starting point to identify these potential elements and generate new hypotheses; functional experiments must be performed for any gene of interest in such signaling pathway, as we have for NPR1 (NPR-A), which inspire us to investigate the modulatory effects of ANP on several types of neuronal functions within the SG may be involved in through its cell surface receptors. The role of ANP in the regulation of neural development may support it as a key regulator for auditory development and regeneration within the cochlear SG. Manipulation of cGMP levels and activation of PKG by activating ANP and receptor signals represent a potential therapeutic approach to support SGN survival as well as enhance regeneration and regrowth of SGN neurites, which promise to be a fruitful area for developing new and effective therapies for hearing impairment.

## Data Availability

The data used to support the findings of this study are available from the corresponding author upon request.
